# Experimental and Finite Element Investigation of Bolted Connections in GFRP Composite Cross-Arms for Energy Distribution Towers

**DOI:** 10.3390/polym18080978

**Published:** 2026-04-17

**Authors:** Burak Talha Kılıç, Eray Baran

**Affiliations:** Department of Civil Engineering, Middle East Technical University, 06800 Ankara, Türkiye; erayb@metu.edu.tr

**Keywords:** bolted connections, composite cross-arm, energy distribution tower, finite element modeling, glass fiber-reinforced polymer

## Abstract

This study investigates bolted connections in open-section glass fiber-reinforced polymer (GFRP) composite cross-arms for 34.5 kV energy distribution towers. Six GFRP angle sections (L50 × 5 to L120 × 12) were tested under tensile loading using a constant edge distance-to-bolt diameter ratio (e/d = 5), and the connection performance was evaluated based on general maximum and deformation-based criteria (4% and 1 mm hole elongation). Connection capacities ranged from 14.65 to 36.68 kN for single-bolt configurations. Results from multi-bolt connections tests indicated strong influence of bolt layout on connection performance. The highest load capacities of 46.45 kN and 45.93 kN were obtained, respectively, with the two-row bolt configuration and staggered configuration. Comparison of the measured load capacities with ASCE/SEI 74-23 predictions revealed significant discrepancies depending on the assumed failure mode of the connection. A simplified finite element model was developed to predict load–displacement response, capturing initial stiffness and overall trends with reasonable agreement, particularly for connections exhibiting similar failure modes. The findings provide a reliable basis for selecting appropriate bolted connection details in open-section GFRP cross-arm systems.

## 1. Introduction

**Figure 1:**
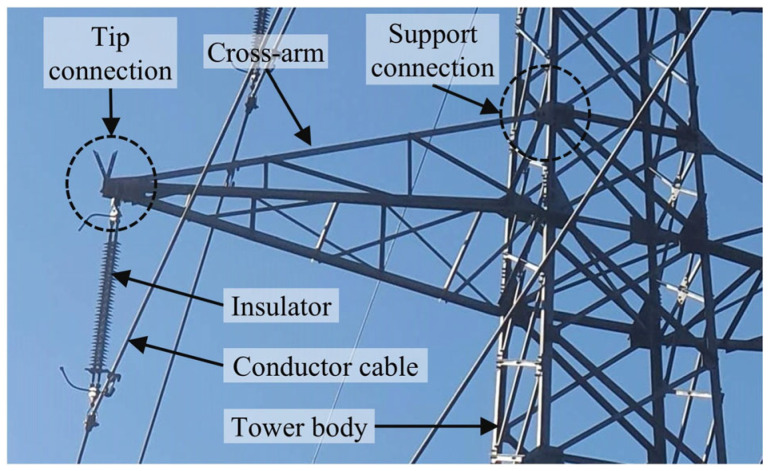
Main components of a typical cross-arm structure in an energy distribution tower.

The structural reliability of power transmission and distribution systems largely depends on the performance of lattice towers, which are used as supporting structures. Such towers are commonly fabricated from steel angle profiles connected by bolted joints, creating rigid structures that are capable of resisting high electrical and mechanical demands. Within these towers, two principal structural components can be distinguished: the tower body, which provides overall stability and the cross-arms, which extend outward from the tower body to support conductor cables and insulator strings. The cross-arms serve a critical dual function; they carry the weight of the conductors while ensuring that sufficient clearance is maintained between energized lines and grounded tower members. As illustrated in Figure 1, the insulator assemblies attached to the cross-arms provide electrical insulation between conductors and structural members, thereby preventing electrical faults.

Among the materials used in power distribution infrastructure, steel has long dominated the construction of towers and cross-arms due to its strength, stiffness, and availability. However, steel components are inherently heavy, which complicates transportation and erection. In addition, steel is highly susceptible to corrosion and may require painting or galvanizing treatments during the lifetime of towers to ensure durability, particularly in aggressive environments. These drawbacks have motivated the search for alternative materials that combine mechanical efficiency with long-term durability. Fiber-reinforced polymer (FRP) composites have gained attention in this regard, offering a unique combination of advantageous properties. FRP members are lightweight, which simplifies installation, while their corrosion resistance substantially lowers maintenance demands. Furthermore, their inherent dielectric properties provide electrical insulation, reducing the required length of insulator strings and the cross-arm itself, thereby allowing more compact tower configurations. It has been shown that sufficiently long GFRP members exhibit superior resistance to flashover and enhance the electrical performance of cross-arm assemblies [1]. These material-level advantages make FRP composites a promising alternative to steel in electricity distribution tower applications.

**Figure 2:**
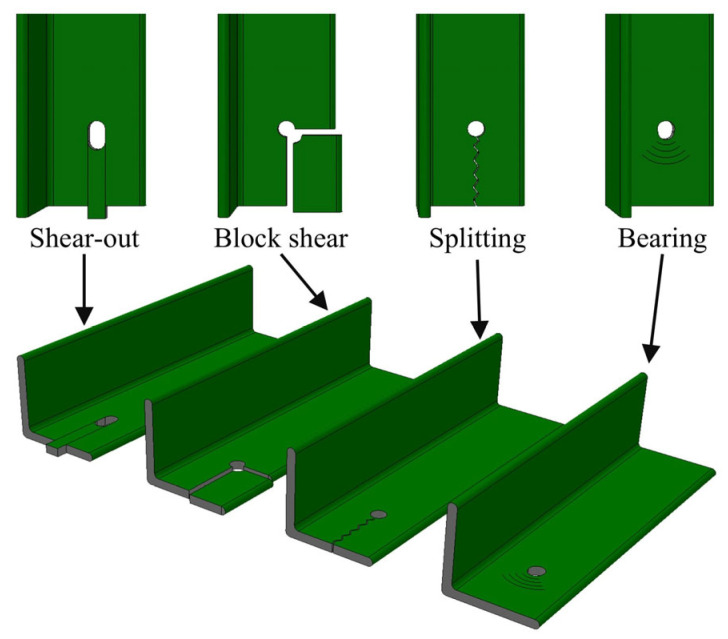
Typical failure modes observed in bolted GFRP angle connections: shear-out, block shear, splitting, and bearing.

Despite the clear material advantages of FRP composites, their structural application is mostly governed by the behavior of member connections. Unlike steel, which is generally treated as an isotropic material exhibiting ductile response, FRP is anisotropic and brittle, making the design of joints considerably more complex. In particular, bolted connections require special attention due to the presence of multiple failure modes interacting with each other. Bolted connections in GFRP members are prone to brittle failure mechanisms such as shear-out, splitting, and block shear, in addition to bearing-type damage (Figure 2). The governing failure mode is highly dependent on parameters such as the edge distance-to-bolt diameter (e/d) ratio and the width-to-bolt diameter (w/d) ratio. These parameters directly influence the level of ductility that the connection exhibits. In critical structural components, brittle failures can compromise system reliability, whereas bearing-type damage is generally favored because it offers a relatively more ductile response. These considerations highlight the importance of continued research into the behavior and optimization of FRP bolted connections, with particular emphasis on enhancing their reliability for use in distribution tower cross-arms.

The application of FRP materials in power transmission and distribution lines is relatively recent. The use of GFRP composites in distribution/transmission infrastructure can be examined from different perspectives, with prior studies broadly grouped into four areas: (1) investigations on GFRP composite cross-arms, including experimental full-scale testing, numerical modeling, and hybrid system evaluations [2,3,4,5,6,7,8,9,10,11]; (2) composite-insulator cross-arms, including both numerical uprating analyses and comprehensive mechanical, electrical, and field monitoring investigations [12,13,14,15]; (3) GFRP applications in transmission and telecommunication towers, including experimental programs, modeling of redundant members, and cost studies [16,17,18,19,20,21]; and (4) time-dependent performance of GFRP members in composite cross-arms, with creep investigations demonstrating the significant influence of stacking sequence on short- and long-term deformation [22,23].

Extensive research has been devoted to bolted joints in FRP materials for several decades. In particular, single-bolt connections have been examined in depth to understand the influence of geometric parameters and fiber layup on structural performance [24,25,26]. These studies consistently demonstrate that the width-to-bolt diameter ratio (w/d) and edge distance-to-bolt diameter ratio (e/d) govern both the load-carrying capacity and the ultimate failure mode of the joint. In addition, factors such as bolt threads, bolt-hole clearance, washers, and tightening torque have been investigated for their effects on connection behavior [26,27,28,29,30,31]. Findings indicate that the use of washers and increased tightening torque enhance joint stiffness and load capacity, whereas the presence of bolt threads bearing directly on the FRP material reduces strength compared to plain shank bolts.

Beyond geometric optimization, researchers have also investigated strengthening single- and multi-bolt GFRP connections using externally bonded glass-fiber sheets (GFS) with different fiber orientations [30,32,33,34,35,36,37]. Such reinforcement has been shown to improve load capacity, stiffness, and ductility while promoting more favorable (bearing-type) failure modes. However, the relative effectiveness of GFS tends to diminish as the e/d ratio increases. Studies on multi-bolt configurations have shown that parameters such as bolt orientation, number of bolts, edge distances, and bolt diameter influence stress distribution and may trigger brittle failure mechanisms [38,39]. Results of several experimental studies on multi-bolt GFRP connections of various types indicate that the number, orientation, and spacing of bolts directly influence the load capacity and the progression of damage [28,40,41,42,43]. Studies on GFRP connections with staggered bolt layouts are rather limited [33,44]. Increasing the number of bolts, rather than solely enlarging the edge distance, has been reported to improve connection efficiency more effectively [42]. Nevertheless, when comparing the experimentally obtained response with design recommendations from existing standards, significant discrepancies have been observed in the e/d ratios associated with each governing failure mode [29,37,44]. Finally, numerous numerical investigations have complemented experimental research on both single- and multi-bolt GFRP joints. Finite element (FE) models incorporating progressive damage criteria have successfully predicted load–displacement responses and failure evolution, providing valuable tools for parametric studies and design development [45,46].

An experimental study by Kılıç et al. [47] investigated the behavior of bolted composite connections and subsequently evaluated the structural performance of composite cross-arms composed of pultruded box sections under different connection configurations. The study demonstrated that connection behavior plays a critical role in governing the global response of composite cross-arms, particularly in terms of load capacity and stiffness. In addition, the findings highlighted the structural feasibility of composite cross-arms as a viable alternative to conventional steel systems in energy transmission applications, considering their favorable mechanical performance and practical advantages.

Although bolted FRP joints have been extensively studied and design provisions are available, limited attention has been given to angle-type GFRP members, particularly for cross-arms in power transmission towers. Current applications largely rely on built-up or closed sections, which introduce complex connection details and increased weight, reducing the advantages of GFRP and limiting practical applicability under field conditions. This highlights the need for a detailed investigation of open-section GFRP profiles as more efficient alternatives. In addition, existing studies do not provide a comprehensive evaluation based on a realistic case, where an initial cross-arm design is developed considering actual tower and line parameters, followed by a detailed structural and geometrical assessment of different connection configurations. Moreover, due to the inherently complex damage mechanisms of composite connections, various methods have been proposed to define maximum load capacity; however, their influence on reported capacity and structural interpretation has not been thoroughly examined. In order to address the identified gaps in the current to GFRP cross-arm literature, a comprehensive investigation is required that integrates realistic structural design considerations with detailed connection-level analysis. Such an approach should not only capture the complex damage mechanisms governing GFRP bolted connections but also evaluate their implications on structural performance under representative loading conditions.

The present study considers the use of open-section angle GFRP profiles for cross-arm applications. Accordingly, an integrated experimental and analytical framework was adopted to examine connection behavior, assess existing design assumptions, and explore practical alternatives for cross-arm systems. The specific objectives and contributions of the study are outlined below.

- To develop a realistic cross-arm configuration based on actual tower and line parameters, providing a practical basis for evaluating GFRP cross-arm systems.
- To introduce the use of open-section angle GFRP profiles for cross-arm structures as a practical and structurally efficient alternative to conventional configurations.
- To experimentally investigate the behavior of single- and multi-bolt connections in angle-type GFRP members, focusing on strength, stiffness, and failure mechanisms.
- To evaluate different capacity definition methods and quantify their influence on reported load capacity, deformation characteristics, and structural interpretation.
- To assess the applicability of a simplified finite element modeling approach, considering its accuracy and limitations with respect to failure modes and connection configurations.
- To establish a mechanism-based and experimentally validated framework for the design of GFRP cross-arm connections, accounting for bolt interaction effects and complex damage mechanisms.

## 2. GFRP Composite Cross-Arm Structure

The proposed GFRP composite cross-arm was developed as a direct replacement for conventional steel cross-arms used in suspension type distribution towers. Its geometry was determined to match the existing steel system in order to ensure structural compatibility and allow installation without modification to the tower body. The prototype tower considered in this study is equipped with six cross-arms. Among these, the center cross-arm is the most critical one due to its longest span length of 1900 mm. Therefore, this cross-arm was selected for the preliminary design.

**Figure 3:**
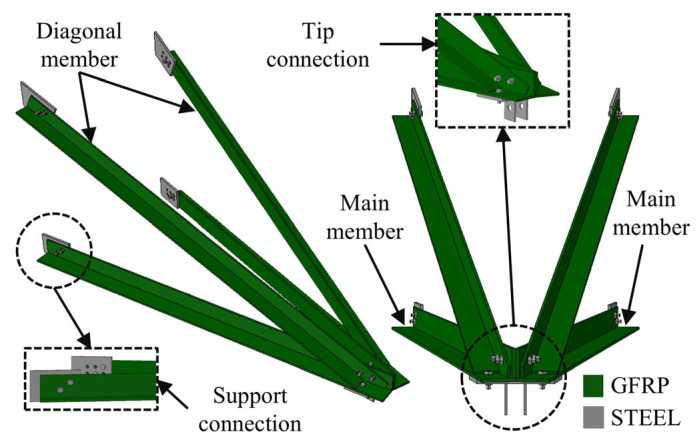
Proposed GFRP composite cross-arm: overall view and connection details.

The proposed composite cross-arm consists of four pultruded GFRP angle profiles. Two of these members are designated as main members, while the other two are diagonal members. The connection of the composite members to the tower is achieved through the existing steel connection plates on the tower body using steel bolts. At the tip region of the cross-arm, where the four composite members intersect and the insulator set is attached, a steel connection plate was designed to provide connection between the composite members through bolts. This plate also enables the attachment of insulator set to the cross-arm tip. The overall view of the proposed GFRP composite cross-arm and the connection details are shown in Figure 3. The use of angle profiles in the proposed design simplifies connection details and eliminates the complex joint configurations typically associated with closed sections. This approach also reduces the likelihood of fiber damage during bolt tightening.

### Critical Loading Conditions Considered for Cross-Arm Design

The design of cross-arms in Türkiye is typically governed by two standards: the technical specification for high voltage energy distribution lines for 34.5 kV systems [48] and the technical specification for tower design of energy transmission lines for 154 kV systems [49]. In the present study, only the latter specification was adopted, as it accounts for both normal (ice-free) and iced conditions for wind-induced forces, thereby providing a more conservative basis for design. This choice also ensures that the proposed composite cross-arm remains applicable to higher-capacity transmission lines in cases where system upgrades are required.

According to TEİAŞ [49], the loads acting on a cross-arm include conductor weights under normal and iced conditions, insulator and hardware weights, the self-weight of the cross-arm, wind effects on all components, unbalanced conductor forces, and broken conductor scenarios. Using a 3/0 AWG Pigeon conductor, the vertical load in normal condition was 0.75 kN, while the load under 100% icing reached 4.66 kN, more than six times the ice-free case. The insulator and installer weights were taken as 0.18 kN and 0.98 kN, respectively, while the self-weight of the cross-arm was calculated as 0.10 kN.

Wind effects were evaluated under both normal and iced conditions. In normal condition, transverse wind loads on the conductor, insulator set, and cross-arm were calculated as 1.77 kN, 0.14 kN, and 0.26 kN, respectively. In iced condition, the projected surface areas of the cross-arm and insulator set were increased by a factor of 1.5, while the wind pressure was reduced. As a result, the wind loads on the cross-arm and insulator decreased; however, the wind force on the iced conductor increased to 2.98 kN, approximately 70% higher than in the normal case.

Unbalanced icing condition was also taken into account in design by considering that the conductor along one span of the cross-arm experiences 100% ice load while the opposite span carries half of this value. Under this condition, horizontal and vertical forces of 4.27 kN and 3.68 kN, respectively, developed at the cross-arm tip. In addition, conductor breakage scenarios were evaluated, where the sudden loss of tensile force in one span resulted in unbalanced loading at the cross-arm tip. The critical load cases defined by the specification [49], the corresponding calculated loads, the adopted safety factors, and the nature of these load cases are summarized in Table 1.

Linear elastic analyses of the prototype tower were then conducted to determine the internal forces in cross-arm members under the computed critical load scenarios. The results showed that the unbalanced icing case (CL5) governed the design, producing maximum member axial forces of 15.39 kN in tension and 16.63 kN in compression. These values were adopted as the governing design forces for evaluating connection capacity of the cross-arm structure for subsequent experimental investigation.

## 3. Experimental Program

The experimental program was developed to evaluate the structural performance of bolted GFRP connections and to establish a design basis for the proposed composite cross-arm. Two groups of tests were performed: single-bolt (Test Group 1) and multi-configuration bolted connections (Test Group 2). In Test Group 1, the single-bolt connection tests were conducted to investigate the influence of different GFRP angle sections on connection behavior while maintaining a constant edge distance-to-bolt diameter (e/d) ratio. These tests also facilitated the selection of the optimum section by comparing the observed connection performance with the design load previously obtained from structural analysis. Subsequently in Test Group 2, multi-configuration bolted connection tests were carried out to examine alternative connection details that could be practically implemented at the tip region of the proposed cross-arm. This group evaluated the effects of section geometry, bolt layout, bolt orientation, and bolt number on connection performance. This experimental investigation enabled the assessment of load transfer mechanisms in more complex configurations, reflecting the demands of actual cross-arm applications. For both test groups, different evaluation criteria commonly used to determine the maximum load-carrying capacity of FRP bolted connections were applied. The effect of test parameters on the connection response was evaluated based on the load-carrying capacity, stiffness, and governing failure mode.

Finally, the experimentally obtained capacities were compared with the design equations provided in ASCE/SEI 74-23 [50], which offers load and resistance factor design (LRFD) provisions for pultruded FRP structures. The level of safety achieved by the selected connection design was assessed with reference to this specification. Such an assessment confirmed that the proposed details are consistent with established FRP design practices and suitable for adoption in full-scale composite cross-arm applications.

### 3.1. GFRP Composite Materials

**Figure 4:**
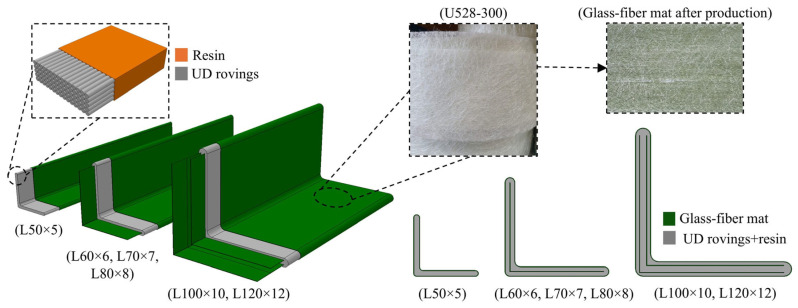
Layup of pultruded GFRP angle sections with UD rovings, resin, and U528-300 glass-fiber mat layers (MİTAŞ Composites, Ankara, Türkiye).

Pultruded GFRP angle profiles used for the fabrication of the test specimens were manufactured by MİTAŞ Composites (Ankara, Türkiye). The composite material consisted primarily of E-glass fibers and polyester resin, accounting for approximately 90% of the total composition by weight. E-glass rovings with a tex value of 4800 were used as the main reinforcement, while nonwoven glass-fiber mats were incorporated to improve transverse properties. The layup followed a skin–core–skin configuration, where the core consisted of unidirectional (UD) longitudinal rovings (0°) impregnated in the resin matrix and the skins were formed by the nonwoven glass-fiber mat layers (Figure 4).

During the pultrusion process, nonwoven mat layers were placed on both the inner and outer surfaces of the angle profiles. Additional mat layers were also embedded within the thickness of the legs of the angle section. The nonwoven glass-fiber mat incorporated in the pultruded profiles was U528-300, with a nominal areal weight of 300 g/m^2^. This mat has a nonwoven structure and consists of continuous glass strands that are randomly oriented and bound together with a binder. The fibers are made of E-CR glass (E-glass corrosion resistant), providing improved chemical durability and dielectric performance compared to conventional E-glass.

**Figure 5:**
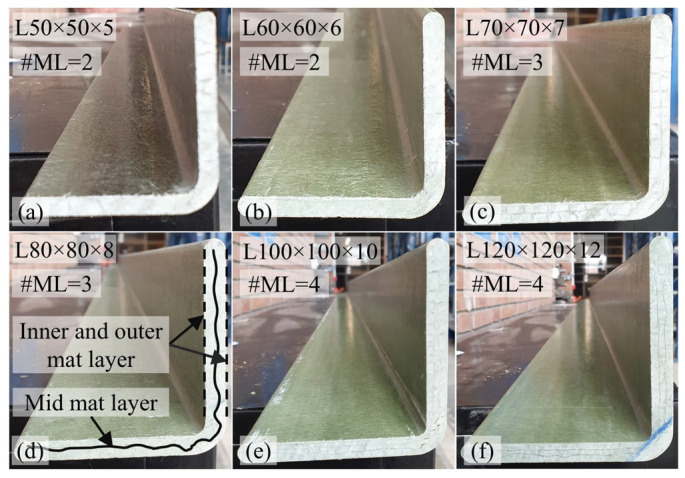
Cross-sectional views of GFRP composite angle sections (#ML: number of mat layers): (**a**) L50 × 5; (**b**) L60 × 6; (**c**) L70 × 7; (**d**) L80 × 8; (**e**) L100 × 10; (**f**) L120 × 12.

Angle profiles with cross-sectional dimensions of L50 × 5, L60 × 6, L70 × 7, L80 × 8, L100 × 10, and L120 × 12 were included in the experimental program. The profiles differed in the number of mat layers: two layers in the L50 × 5 profile; three layers in the L60 × 6, L70 × 7 and L80 × 8 profiles; and four layers in the L100 × 10 and L120 × 12 profiles. Cross-sectional views of the profiles, together with the corresponding number of mat layers, are presented in Figure 5. In order to determine the tensile properties of the GFRP material, five coupon samples were cut from angle profiles and tested. The axial tensile tests were performed in accordance with EN ISO 527-4:1997 [51]. The results indicated an average tensile strength of 475.4 MPa (±19.6 MPa), an elastic modulus of 38.4 GPa (±1.0 GPa), and an ultimate strain at failure of 1.28% (±0.05%). In addition, tensile modulus and tensile strength in the transverse direction were provided by the manufacturer, obtained through testing in accordance with EN ISO 527-4:1997 [51], with reported values of 7 GPa and 75 MPa, respectively.

### 3.2. Connection Specimen Details

**Figure 6:**
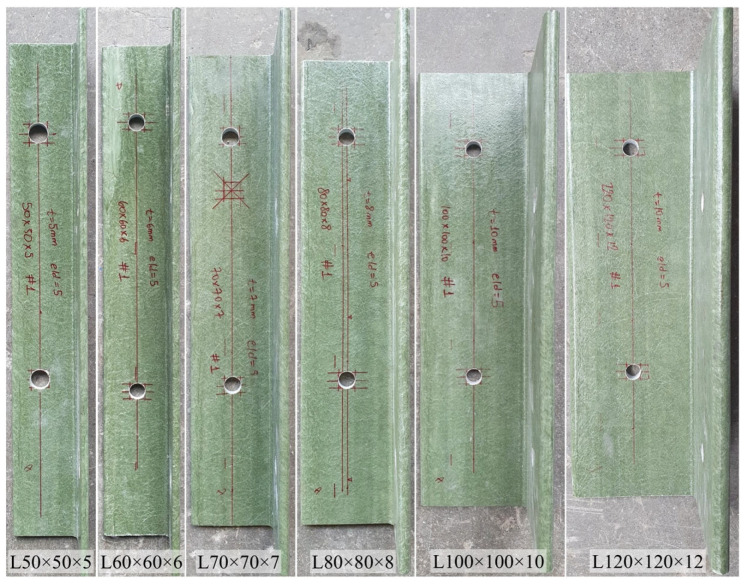
Test Group 1 specimens with sizes ranging from L50 × 5 to L120 × 12 prior to testing.

The bolted connection tests were conducted in two groups. In Test Group 1, single-bolt connection specimens were tested using profiles ranging from L50 × 5 to L120 × 12, with a constant edge distance-to-bolt diameter (e/d) ratio. The strength and stiffness of bolted GFRP connections depend strongly on both the geometry of the connection and the section properties. Among these parameters, the e/d ratio plays a critical role in determining the failure mode. Depending on the magnitude of this ratio, connection may suffer from a brittle failure due to shear-out or exhibit a more ductile bearing failure. Current design documents, including ASCE/SEI 74-23 [50] and CEN/TS 19101:2022 [52], also promote bearing failure in bolted GFRP connections. Specifically, ASCE/SEI 74-23 [50] and CEN/TS 19101:2022 [52] suggest minimum e/d ratios of 4 and 2.5, respectively, in an attempt to prevent a brittle shear-out failure. It should be noted that previous experimental studies have proposed higher e/d limits than these code-specified values, such as 5 and 8 [24,40]. For bearing strength testing in bolted GFRP connections, ASTM D953-10 [53] and ASTM D953-19 [54] specify an e/d ratio of 6. Considering these recommendations and the intended geometry of the proposed cross-arm, an e/d ratio of 5 was adopted in the present study. The bolt diameter was set to 12 mm, corresponding to an edge distance of 60 mm for all single-bolt connection specimens in Test Group 1. For each profile type, four identical specimens were fabricated, producing a total of 24 specimens. The pultruded profiles, supplied in 6 m lengths, were cut to size using a diamond saw designed for composite materials. Bolt holes with 13 mm diameter were drilled using drill bits specifically manufactured for FRP sections to minimize delamination and fiber breakout. Representative images of the single-bolt connection specimens prior to testing are presented in Figure 6.

**Figure 7:**
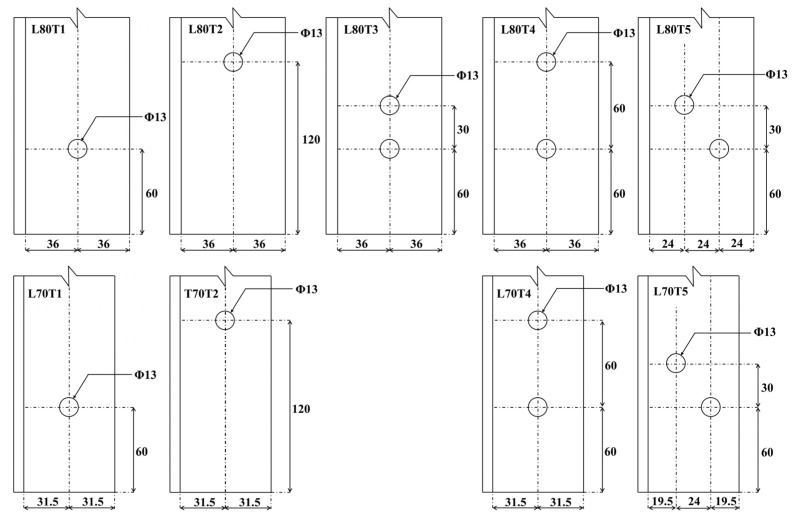
Geometrical details of Test Group 2 specimens (dimensions are in mm).

**Figure 8:**
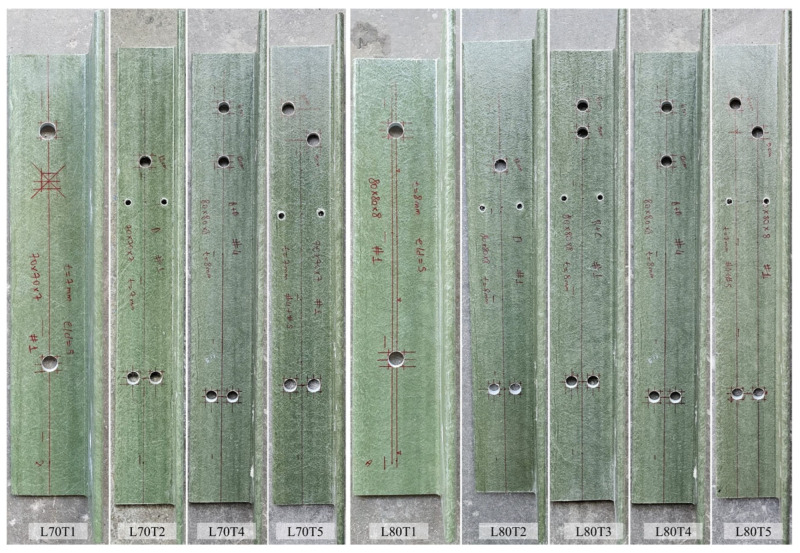
Test Group 2 specimens (L80 × 8 and L70 × 7) prior to testing.

In the second group of the experimental program, multi-configuration bolted connection tests were carried out on L80 × 8 and L70 × 7 profiles to investigate the effect of different bolt arrangements. Five different bolt configurations were designed for the L80 × 8 section, while four bolt configurations were considered for the L70 × 7 section. A schematic representation of the bolt configurations utilized in these specimens is shown in Figure 7. For each configuration, four specimens were fabricated, resulting in a total of 36 specimens. Images of the multi-configuration bolted connection specimens in Test Group 2 prior to testing are provided in Figure 8.

### 3.3. Test Setup and Instrumentation

**Figure 9:**
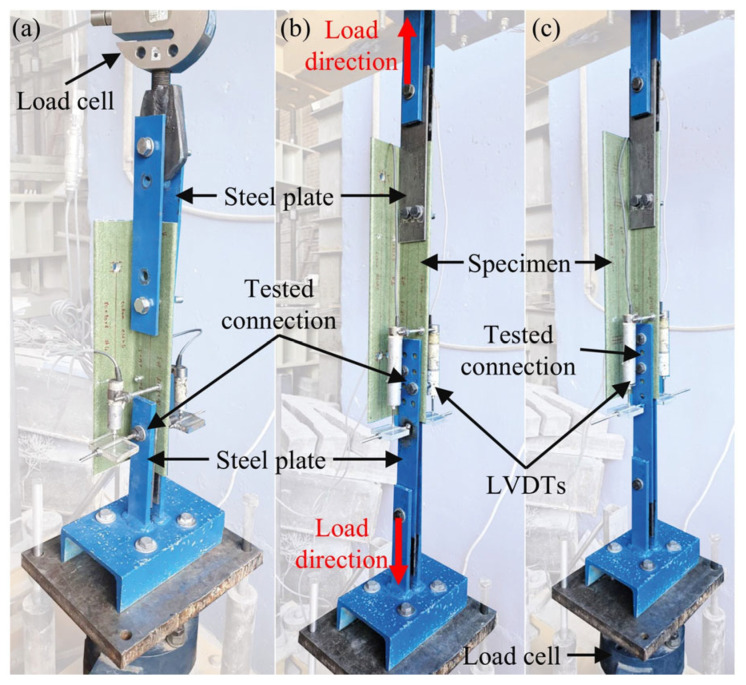
Loading setup used for connection tests: (**a**) Test Group 1; (**b**) T5 configuration in Test Group 2; (**c**) T2, T3, and T4 configurations in Test Group 2.

The experimental setup used for the connection tests is shown in Figure 9. The tests were conducted under monotonic axial tension loading using an electro-mechanical loading frame operated in displacement control at a rate of 0.2–0.3 mm/min. To prevent eccentricity and unintended rotations within the connection, the loaded leg of each GFRP angle profile was clamped between steel plates in a double-lap joint configuration. The load applied on the specimens was measured using a 200 kN capacity load cell. Connection slip was monitored by two linear variable differential transformers (LVDTs) mounted on opposite sides of the specimen. For this purpose, 10 mm capacity LVDTs were used in Test Group 1 specimens while 50 mm capacity LVDTs were used in Test Group 2 specimens. The average of two LVDT readings was reported as the connection slip for each specimen. Data from the load cell and displacement transducers were collected through a multi-channel data acquisition system, which enabled synchronized logging and real-time visualization of the test data.

In bolted GFRP connections, the tightening torque applied to fasteners can influence the measured load-carrying capacity of the connection. Up to a certain threshold, increasing the tightening torque enhances the apparent connection strength [28,31]. To eliminate this secondary effect and ensure that the experimental results reflect the behavior of a pure bearing-type bolted connection, all test bolts were installed under hand-tightened condition without an applied torque. On the other hand, the bolts used to attach the specimen to the testing frame were fully tightened to provide adequate restraint and prevent unintended movements during loading. Previous studies have shown that direct contact between the bolt threads and the FRP material around the hole surface can significantly reduce the connection capacity [27,30]. This effect was mitigated by ensuring that the bolt threads were not in direct bearing contact with the GFRP material in the test specimens.

### 3.4. Test Group 1: Single-Bolt Connection Tests

**Figure 10:**
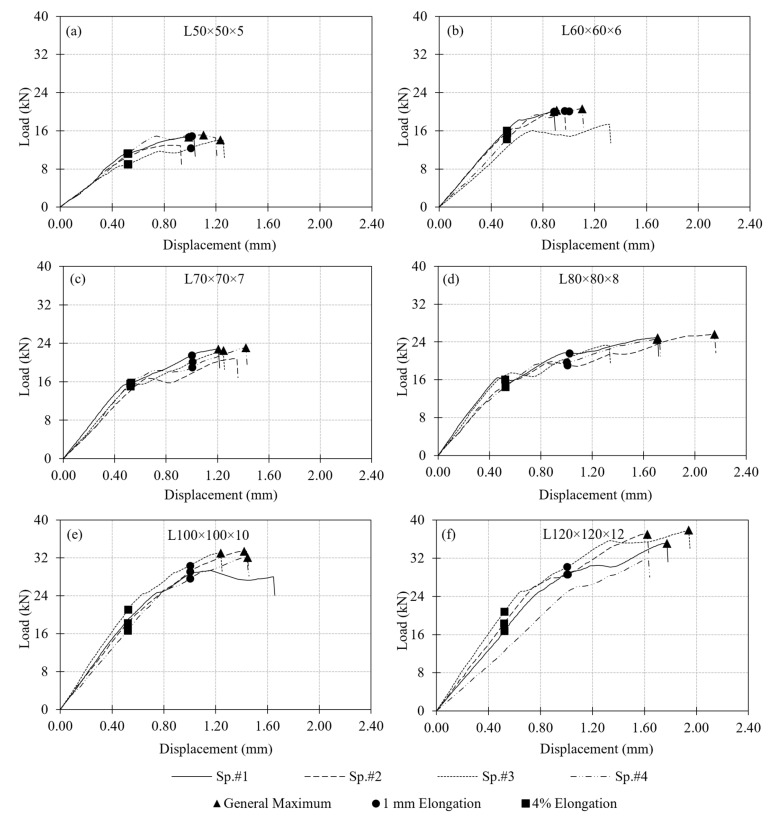
Load–displacement responses of Test Group 1 specimens: (**a**) L50 × 5; (**b**) L60 × 6; (**c**) L70 × 7; (**d**) L80 × 8; (**e**) L100 × 10; (**f**) L120 × 12.

A total of 24 single-bolt connection tests were performed in Test Group 1 using six different cross-sections ranging from L50 × 5 to L120 × 12, each prepared with a constant e/d ratio of 5. This ratio resulted in an edge distance of 60 mm for all specimens. For each section size, four specimens were tested under monotonic tensile loading. Out of these four tests, one outlier result was disregarded when calculating the average load capacity for each section size. Specimen response was evaluated mainly based on the measured load–displacement behavior. The displacement used in the evaluation is the relative displacement between the GFRP angle section and the connection bolt in the direction of the applied loading. The load–displacement curves for all section sizes are presented in Figure 10 with the corresponding test results summarized in Table 2. In the table, the outlier specimen, which was disregarded when calculating the average load capacity for each section size, is marked with an asterisk.

Examination of the load–displacement curves in Figure 10 reveals that the observed increase in load-carrying capacity is associated with both the increase in cross-sectional thickness and the incorporation of additional mat layers. Although individual contributions of these two parameters cannot be isolated, their combined effect leads to a noticeable improvement in the overall connection response. While the initial stiffness of the connections is relatively similar across all section sizes, clear differences emerge beyond the initial linear region. Specimens with larger section sizes exhibit a more gradual stiffness degradation, maintaining load-carrying capability over a wider displacement range. In contrast, smaller sections tend to show a more pronounced reduction in stiffness following the onset of damage. This behavior is further reflected in the smoother post-peak response observed in thicker sections, indicating a more stable progression of damage. Overall, the results suggest that the combined influence of increased GFRP thickness and mat content enhances not only the load capacity but also the deformation tolerance of the connection response.

**Figure 11:**
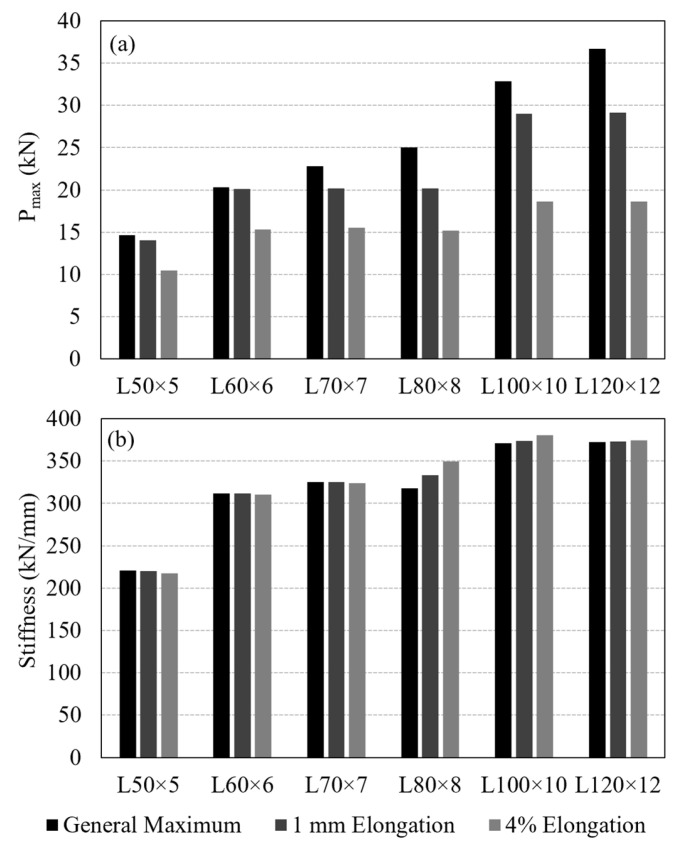
Test Group 1 results determined based on different criteria: (**a**) load capacities; (**b**) service stiffnesses.

A critical issue in evaluating the performance of a bolted GFRP connection is the criterion used to determine load-carrying capacity from the measured load–displacement curve. Several load capacity definitions exist in the literature. Mottram and Zafari [55] emphasized the complexity of this problem and summarized the common approaches, including: (i) the maximum recorded load (ASTM D953-19), (ii) a displacement-based criterion such as 4% hole elongation (ASTM D953-10), (iii) load at the first peak, and (iv) the point of visible or audible cracking. In this study, three criteria were adopted to determine the connection load capacity for comparison: the general maximum (ASTM D953-19), the 4% hole elongation (ASTM D953-10), and the 1 mm hole elongation criteria. With the 13 mm bolt hole diameter used in the test specimens, the 4% hole elongation corresponds to 0.52 mm displacement. The capacities computed by following these three criteria are indicated in Figure 10 on the experimental load–displacement curves and summarized in Table 3. A comparison of load capacities determined based on the different criteria is provided in Figure 11a.

**Figure 12:**
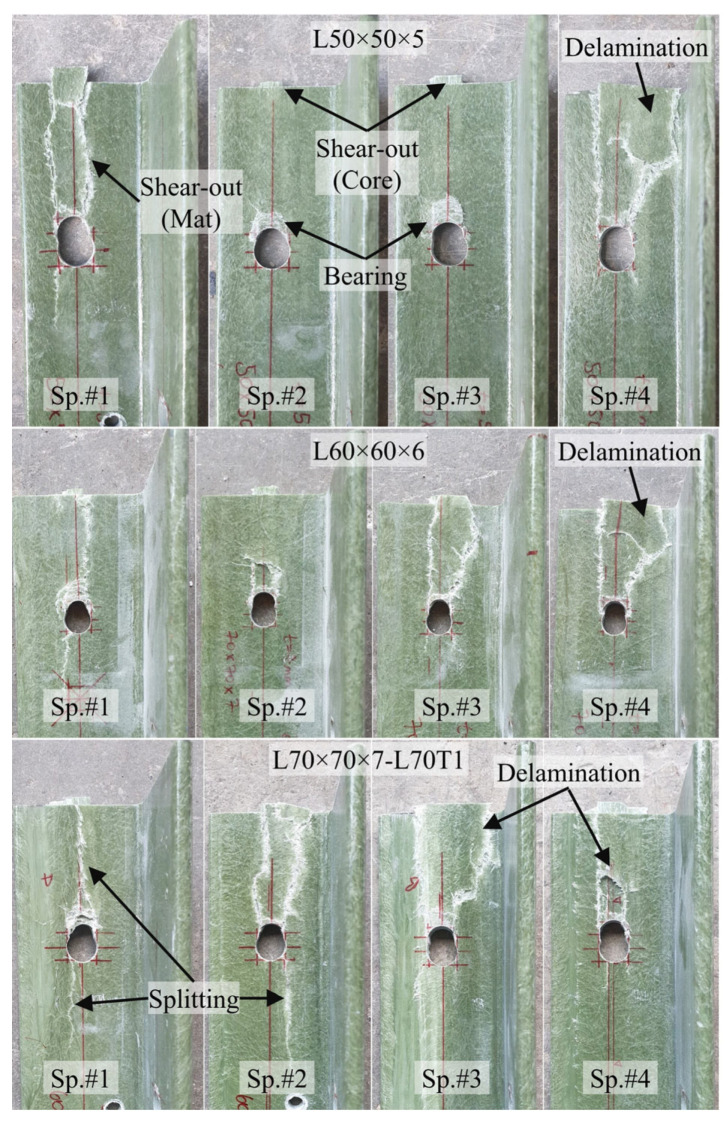
Damage observed in Test Group 1 specimens at the end of testing: L50 × 5, L60 × 6, L70 × 7.

**Figure 13:**
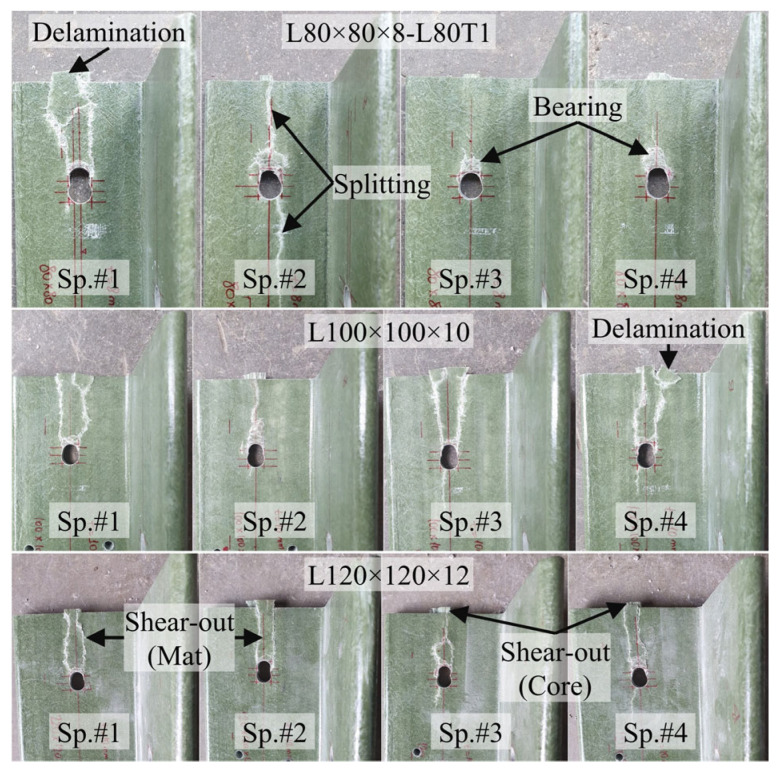
Damage observed in Test Group 1 specimens at the end of testing: L80 × 8, L100 × 10, L120 × 12.

Examination of the results revealed that the adopted e/d = 5 configuration did not lead to pure bearing or pure shear-out failures. Majority of the specimens initially developed bearing deformations around the bolt hole, followed by stiffness degradation and eventually shear-out failure at higher loads. The failure patterns observed during the load tests are further examined and discussed in detail in Section 3.6. Photographs showing the condition of the GFRP members at the end of load testing also depict the signs of GFRP shear-out and bearing around bolt holes in majority of the specimens (Figure 12 and Figure 13).

When the average load capacities for Test Group 1 specimens are compared, the general maximum criterion shows a consistent increase in capacity from L50 × 5 to L120 × 12. However, the 4% elongation and 1 mm elongation criteria do not indicate a clear trend. Differences among the load capacities determined using the three criteria explained above are relatively small for the GFRP thicknesses of 5 mm and 6 mm (Figure 11a), however the differences become more pronounced with further increase in the thickness. This can be attributed to the larger displacement values observed in the specimens with thicker GFRP profiles than those with thinner profiles.

Another observation valid in Figure 11a is that there is a notable increase in load capacities determined based on the 4% elongation and 1 mm elongation criteria between L50 × 5 and L60 × 6, as well as between L80 × 8 and L100 × 10. As mentioned earlier, these correspond to the section sizes that the number of interior mat layers in the cross-section increases. No interior mat was present in L50 × 5; there was a single layer of interior mat in L60 × 6, L70 × 7 and L80 × 8; and two layers of mat were present in L100 × 10 and L120 × 12 (Figure 4). Each time the number of interior mat layers in the cross-section increases, the 4% elongation and 1 mm elongation criteria indicate an increase in load capacity, whereas no appreciable increase occurs among the specimens with the same number of mat layers. This behavior may be attributed to a more uniform distribution of stresses in the bearing region as both the cross-sectional thickness and the number of mat layers increase. The presence of additional mat layers, together with increased thickness, likely enhances the ability of the material to redistribute local stresses around the bolt hole, leading to improved connection performance. An increase in bearing strength with decreasing d/t ratio, where d is the bolt diameter and t is the thickness of the connected GFRP member, has also been reported in the literature [55], supporting the trend observed in the present test results.

Service stiffness was also evaluated as a key design parameter of the tested connections. Following common practice, service stiffness of the specimens was defined as the secant stiffness measured at 60% of the maximum load. The calculated values are presented in Figure 11b and summarized in Table 3. For a given GFRP section size, the service stiffness values are found to be nearly identical across all three criteria. When comparing across the section sizes, trends similar to the strength results are observed. Service stiffness increases noticeably from L50 × 5 to L60 × 6 and from L80 × 8 to L100 × 10. Again, specimens with the same number of mat layers exhibited similar stiffness values.

To further evaluate the effect of cross-section thickness and the number of mat layers, shear-out strength was calculated for each specimen using Equation (1). In this equation F_sh_ is the shear strength, P_max_ is the computed maximum load capacity, t is the thickness of the specimen, e is the edge distance, d_n_ is the bolt hole diameter. Results indicate that the increase in shear-out strength remains almost constant and does not follow the same trend as the increase in load-carrying capacity (Table 2). This observation indicates that the increase in load capacity is driven by section thickness rather than the presence of additional mat layers. The shear-out strength, F_sh_, of the GFRP material was determined to be 29.95 MPa, calculated as the average of the values presented in Table 2.

(1) Fsh=Pmax2e−dn2t

As mentioned earlier, the maximum tensile force on the prototype cross-arm members is determined to be 15.39 kN. Based on the load capacities obtained from single-bolt connection tests in Test Group 1, as well as by considering the overall economy, the L80 × 8 and L70 × 7 sections were identified as the most suitable candidates, as they provide the required load capacity with an additional safety factor. Therefore, these two GFRP sections were further investigated in the multi-configuration bolted connection tests in Test Group 2 presented in the following section.

### 3.5. Test Group 2: Multi-Configuration Bolted Connection Tests

**Figure 14:**
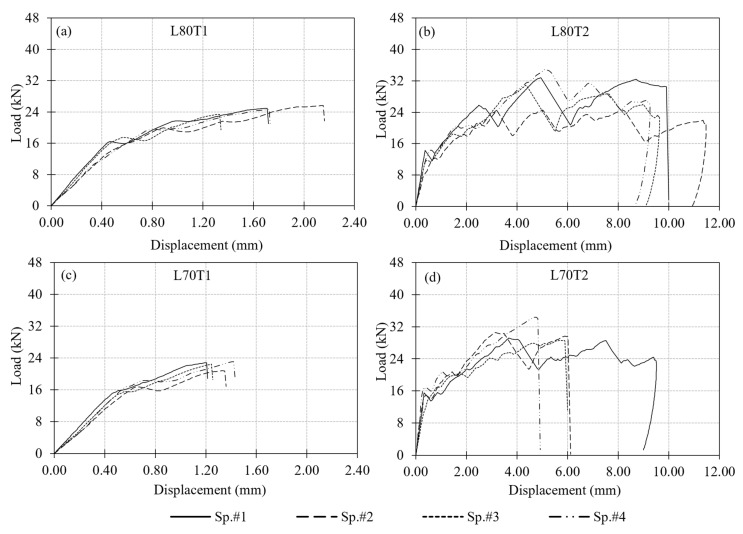
Load–displacement responses of single-bolt connection specimens in Test Group 2: (**a**) L80T1; (**b**) L80T2; (**c**) L70T1; (**d**) L70T2.

**Figure 15:**
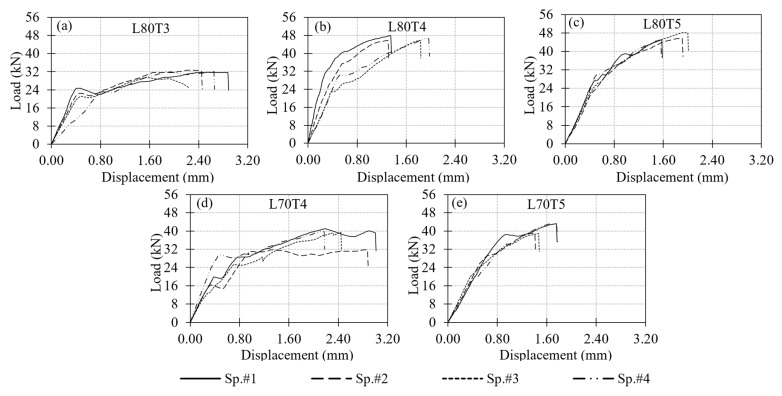
Load–displacement responses of double-bolt connection specimens in Test Group 2: (**a**) L80T3; (**b**) L80T4; (**c**) L80T5; (**d**) L70T4; (**e**) L70T5.

**Figure 16:**
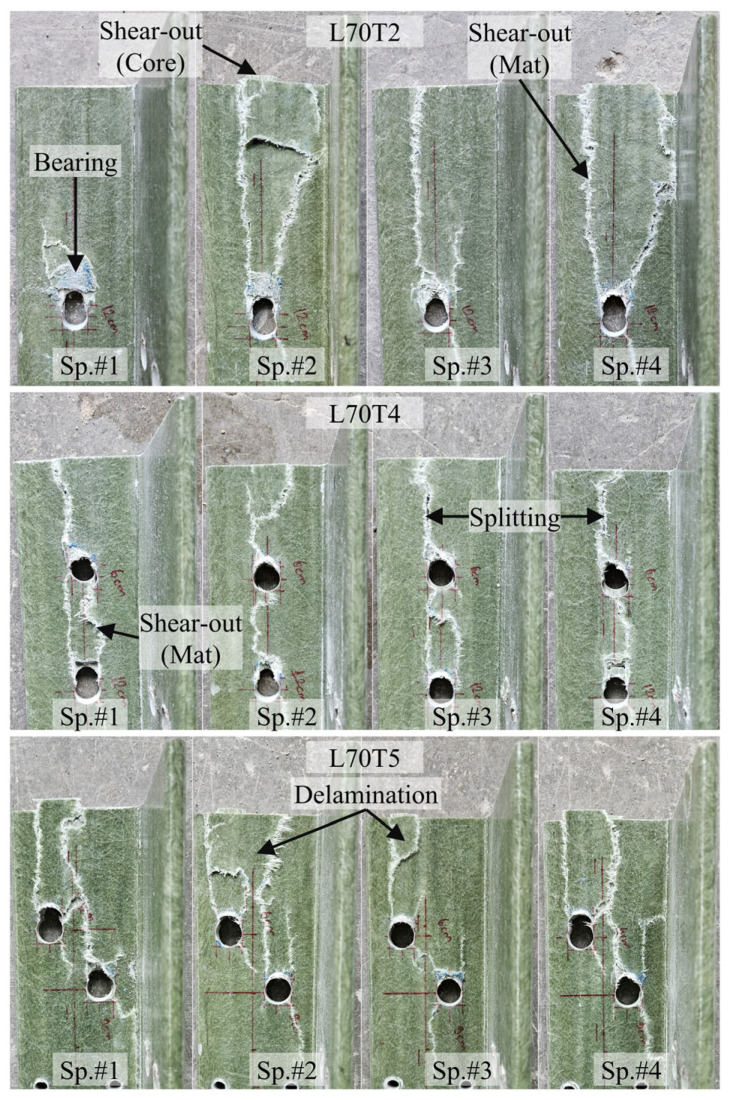
Damage observed in Test Group 2 specimens at the end of testing: L70 × 7.

**Figure 17:**
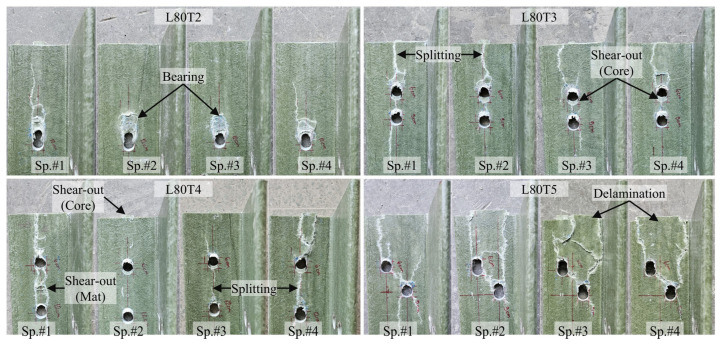
Damage observed in Test Group 2 specimens at the end of testing: L80 × 8.

**Figure 18:**
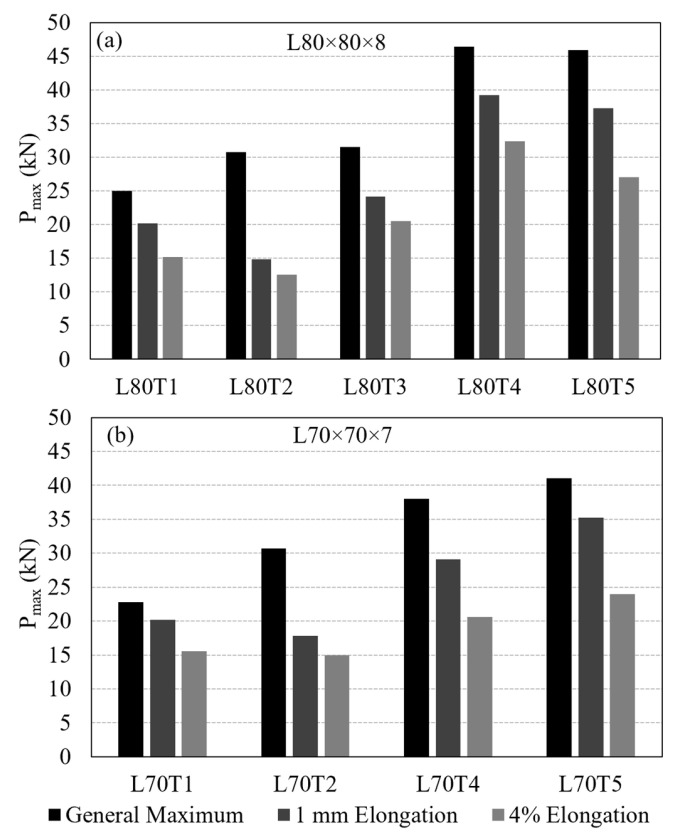
Load capacities of Test Group 2 connections determined based on different criteria: (**a**) L80 × 8; (**b**) L70 × 7.

A total of 22 tests were performed on the multi-configuration GFRP bolted connection specimens in Test Group 2. In this group, T1 and T2 correspond to single-bolt connections, while T3, T4, and T5 represent the double-bolt connection layouts. Since the L70T1 and L80T1 configurations were previously tested in Test Group 1, those tests were not repeated. The failure modes of these reference connections were reported earlier in Figure 12 and Figure 13, respectively, and are adopted here for comparison. The experimental load–displacement results for the single-bolt connections are presented in Figure 14, and those for the double-bolt configurations are given in Figure 15. Damage patterns observed in Test Group 2 specimens at the end of load testing are presented in Figure 16 and Figure 17. A detailed evaluation of the failure behavior of the GFRP connections is presented in Section 3.6. The maximum load-carrying capacities of the tested connections were evaluated using three different criteria similar to single-bolt tests (Test Group 1): the general maximum (ASTM D953-19), the 4% hole elongation (ASTM D953-10) and the 1 mm hole elongation criteria. Relation among the load capacities determined by these three criteria is illustrated in Figure 18 and summarized in Table 4.

The load–displacement response of the T2 type connections (i.e., connections L80T2 and L70T2) differed significantly from the rest of the specimens tested in the present study (Figure 14b,d). These connections exhibited significant levels of displacement with fluctuations in load, which indicate damage accumulation and a series of local material failures. These connections have an e/d ratio of 10 and the overall load-carrying capacity was primarily governed by bearing behavior, while more advanced damage stages involved multiple interacting mechanisms. Compared to other configurations, brittle shear-out failure was only observed in a limited number of specimens. For the T2 type connections, there is a marked difference between the load capacities determined from the general maximum criterion and those determined based on the other two evaluation criteria. The reason for this is the fact that the general maximum load capacity occurs at relatively large displacements and the load levels at the 4% and 1 mm elongations remain considerably small.

Comparison of the single-bolt specimens tested in Test Group 1 (i.e., connection types L80T1 and L70T1) and their counterparts tested in Test Group 2 (i.e., connection types L80T2 and L70T2) is provided in Figure 14. Connections L80T2 and L70T2 exhibited higher load capacities and displacement levels than their counterparts (i.e., connections L80T1 and L70T1) due to increased e/d ratio. The increase in load capacity is 23% and 34%, respectively, for L80 × 8 and L70 × 7 GFRP section sizes. For both section sizes, increasing the e/d ratio from 5 to 10 changed the governing failure mode from shear-out to bearing and led to a more ductile response with higher load capacity.

It is worth noting that connection L80T4 combines the bolt configurations of connections L80T1 and L80T2. However, the load capacity of connection L80T4 is 17% lower than the sum of the load capacities of connections L80T1 and L80T2. The reduction in the expected load capacity of connection L80T4 can be attributed to the interaction between the two bolts. As evident in the plots, the maximum load levels for connections L80T1 and L80T2 occur at different displacement levels. For this reason, summing the load capacities of these two connections does not yield an accurate estimate of the capacity of connection L80T4. The behavior explained here is also valid among specimens L70T1, L70T2 and L70T4 with the load capacity of connection L70T4 being 29% lower than the sum of the capacities of connections L70T1 and L70T2. A similar relationship is also observed for the T1 and T4 connection types. The T4 configuration utilizes two bolts, each with the same edge distance as in the T1 configuration. Therefore, the load capacity of the T4 connections is expected to be approximately twice that of the T1 configuration. However, the experimental results show that the load capacities of the L80T4 and L70T4 connections are 7% and 17% lower than the expected doubled capacities of their T1 counterparts, respectively. The primary reason for this reduction is consistent with earlier observations, specifically the bolt interaction effects that develop due to the relatively small spacing between adjacent bolts. These interactions lead to the formation of complex and interacting damage mechanisms within the composite material, which reduce the load-carrying capacity below the expected values.

Among the double-bolt connections whose load–displacement curves are presented in Figure 15, L80T4 and L80T5 exhibited similar maximum load levels. Load capacities of these connections are approximately 85% higher than the single-bolt L80T1 type connection. In contrast, the L80T3 configuration exhibited a 32% lower capacity than the L80T4 and L80T5 connections. This reduction is attributed to the unfavorable bolt layout in L80T3, where the second bolt had an edge distance of only 30 mm. This reduced clearance promoted shear-out initiation around the second bolt and limited the overall capacity.

When the three criteria used to determine the connection load capacity are compared, the general maximum criterion consistently produced the highest capacities, while the 4% hole elongation criterion yielded the most conservative values. Except for the L80T2 connection, the difference between the capacities determined by each criteria becomes higher as the connection strength increases. For example, the general maximum capacity is 70% and 44% higher than the capacity obtained using the 4% elongation criterion for L80T5 and L80T4, respectively.

Among the double-bolt connection configurations tested with L80 × 8 and L70 × 7 GFRP cross-sections in this group, the T5 connection configuration demonstrated superior structural performance with a compact bolt configuration. The staggered bolt layout utilized in this connection allowed for increased edge distances for the bolts. As a result, a practical and efficient geometric arrangement was obtained for member-to-member and member-to-tower joints in the proposed GFRP cross-arm design. A linear elastic analysis of the prototype cross-arm revealed that the maximum tensile force generated under the factored critical loading condition is 15.39 kN. It should be noted that this force already includes a load factor of 1.5. The experimentally determined capacities of 45.93 kN (for L80T5) and 41.05 kN (for L70T5) correspond to an overall safety factor of 4.48 and 4.00, respectively.

### 3.6. Failure Mechanisms and Damage Development in GFRP Connections

Bolted connections in pultruded GFRP profiles are commonly associated with failure modes such as bearing, shear-out, block shear, and splitting, as illustrated in Figure 2. However, the experimental observations of the present study indicate that the damage development cannot be represented by a single dominant failure mode. Instead, the failure behavior is region-dependent, with different damage characteristics observed in the GFRP core region and the mat-rich layers.

In this study, the GFRP core region refers to the fiber-dominated part of the pultruded profile, primarily composed of longitudinal fibers and resin, which governs the main load-carrying behavior of the connection. In contrast, the mat-rich layers correspond to the outer and intermediate regions containing nonwoven glass-fiber mats, which are more prone to damage mechanisms such as splitting and delamination. Layer-dependent failure characteristics have been reported in recent studies [29,32,37], where different material constituents within composite sections exhibited different dominant damage mechanisms. Furthermore, progressive and interacting damage mechanisms under in-plane loading have been previously highlighted by Russo [56,57], supporting the multi-stage nature of failure in composite connections.

The observed failure modes for all single-bolt specimens in Test Group 1 are summarized in Table 5. As shown, bearing damage was consistently observed in both the GFRP core and mat-rich layers in all specimens, indicating that it governs the initial response of the connection. In the GFRP core region, the failure behavior was uniform across all configurations, where bearing damage was followed by shear-out failure. This indicates that the ultimate capacity of the connection is governed by the fiber-dominated region. In contrast, the mat-rich layers exhibited multiple and coexisting damage mechanisms, including splitting, defined as crack propagation along or opposite to the loading direction; delamination, referring to separation between composite layers; and shear-out-type rupture, characterized by tearing from the hole toward the free edge. These mechanisms were observed either individually or in combination. For example, a combination of splitting, delamination, and shear-out-type damages occurred in L50 × 5 (Sp. #1), L70 × 7 (Sp. #2), and L100 × 10 (Sp. #3–4).

The multi-configuration bolted connection specimens in Test Group 2 exhibited a more complex failure behavior than the single-bolt ones due to the interaction between adjacent bolt holes, as summarized in Table 6. While bearing damage remains present at the bolt holes, the extent of this damage depends on the bolt arrangement. In configurations with sufficient edge distance (e.g., L80T1, L80T4, and L80T5), bearing followed by shear-out was observed in the core region, whereas in configurations with reduced edge distance (e.g., L80T3), bearing development was limited and the failure was governed by shear-out. In this configuration, although the first bolt has an edge distance twice that of the second bolt, the overall connection capacity is governed by the weakest location. This experimental observation also supports the capacity prediction approach of ASCE/SEI 74-23, in which the overall connection capacity is governed by the weakest failure mode within the connection. Therefore, the experimentally observed behavior, where the capacity is controlled by the most critical damage mechanism, is consistent with this design assumption. Furthermore, although the T2 configuration was tested with a relatively high edge distance-to-bolt diameter ratio (e/d = 10), shear-out failure was still observed in the GFRP core region following bearing damage in some L80 and L70 specimens. This indicates that brittle shear-out failure is possible even with relatively high e/d ratios. This observation is consistent with findings reported in the literature [40] and highlights the critical importance of connection design in controlling failure behavior in composite structures.

In the mat-rich layers, the dominant failure mode in multi-bolt connections was splitting, where cracks propagated along the loading direction and frequently linked adjacent bolt holes. This connected splitting behavior was consistently observed in both L70 and L80 series. Delamination was also widely observed, particularly in configurations with more extensive damage (e.g., L80T5 and L70T5), and often occurred together with splitting. Compared to single-bolt connections, shear-out-type failure in the mat-rich layers becomes less pronounced in multi-bolt configurations. While it was observed in some cases (e.g., L70T1 and L70T2), it remained localized. In configurations with strong interaction effects (e.g., L80T5 and L70T5), shear-out was largely suppressed, and the failure was governed by distributed splitting and delamination.

Overall, the results indicate a clear transition from localized damage in single-bolt connections to interaction-driven and distributed damage in multi-bolt configurations, where splitting and delamination become dominant. This highlights the critical role of bolt interaction in governing the failure behavior of pultruded GFRP connections.

### 3.7. Connection Load Capacities According to ASCE 74-23 Provisions

The ASCE/SEI 74-23 guideline provides detailed procedures for predicting connection capacity as a function of e/d ratio, bolt orientation, and the number of fasteners. According to the specification, in multi-row bolted connections, all bolts are assumed to fail through the same governing mode, and the capacity of the connection is controlled by the weakest failure mechanism. In the present study, the nominal connection capacities were calculated using the damage modes and equations prescribed by the guideline without applying any safety factors and these values are reported as P_ASCE_ in Table 7. As a second approach, connection load capacities were calculated using the ASCE/SEI 74-23 equations but adopting the experimentally observed failure modes. Load capacities determined with this second approach were denoted as P_ASCE-exp_ in Table 7.

The equations of ASCE/SEI 74-23, when used with failure modes defined according to the guideline-specified e/d ratio limits, grossly overpredict the load capacity of the majority of connections. The level of over prediction is as large as 53%, leading to non-conservative estimates. Using the same equations but adopting the failure mode determined from the experiments resulted in a noticeable improvement in prediction accuracy. In this case, load capacities of all connections were underpredicted with the level of under prediction changing between 11% and 31%.

The simplified equation given in Equation (2) is provided for predicting the shear-out capacity of GFRP bolted connections. In this equation, t is the GFRP thickness, e_n_ is the clear edge distance, F_sh_ is the shear strength of GFRP material. F_sh_ is determined from load testing of single-bolt connections failing in shear-out in Test Group 1.

(2) $$P_{shear-out}=2e_{n}tF_{sh}$$

As evident in Table 7, Equation (2) provides highly accurate predictions of load capacities for the specimens that exhibited shear-out failure, even in complex multi-bolt connection details. The level of prediction error changes between 4% and 12%. The ASCE/SEI 74-23 design document uses Equation (3) to calculate the bearing capacity of the bolted connections. In this equation, d_b_ is the bolt diameter, t represents the section thickness, and F_b_ denotes the bearing strength of the composite angle material. Predictions for the T2 connection type are intentionally omitted from Table 7. This is because Equation (3) follows the same bearing capacity expression used in the other two prediction approaches, resulting in fully overlapping predicted values with the corresponding bearing capacity estimates when assessed against the experimental test data.

(3) $$P_{bearing}=d_{b}tF_{b}$$

In the literature, minimum e/d ratio limits were recommended as 5 by Martins et al. [40] and 6 by Rosner and Rizkalla [24]. Identical e/d limits of 6 were also adopted in bearing strength test standards [53,54]. In contrast, ASCE/SEI 74-23 specifies a lower design limit of e/d = 4 for defining bearing-governed failure modes. Examination of the results in Table 7 shows that the code provisions tend to overpredict the connection capacity by interpreting failure as bearing-controlled, whereas the actual experimentally observed failure mode was brittle shear-out. If the failure mode is incorrectly idealized, the risk to structural safety becomes substantial due to the brittle nature of shear-out failure and the limited deformation capacity of GFRP angle sections.

Furthermore, safety evaluation within the LRFD framework shows that the code-specified resistance factors would amplify this non-conservative outcome if explicitly included. The resistance factor for shear-out failure is given as 0.45 in ASCE/SEI 74-23, while the factor for pin-bearing is specified as 0.65. Because the experimental capacities are closer to shear-out-controlled predictions than to bearing-based estimates, adding these resistance factors to the comparative results would further distance the code-predicted capacities from the experimental values and produce a more non-conservative design interpretation.

The study demonstrates that e/d ratio limits for GFRP bolted connections are strongly dependent on material properties, composite composition, and the reinforcement architecture of the angle section. The results also confirm that commonly assumed fixed e/d limits may lead to non-conservative capacity interpretation, highlighting the ongoing need for a broader and more reliable experimental basis to define e/d limits.

## 4. Finite Element Analysis of GFRP Connections

Finite element (FE) investigation of the proposed GFRP connections in Test Group 2 was performed by utilizing L80 × 8 angle section to complement the experimental investigation. A simplified modeling strategy was adopted, in which a hinge-based connector formulation was first calibrated using the results of the L80T1 single-bolt connection specimen. Following validation, the same hinge model was implemented in the FE simulations of the remaining configurations (L80T2–L80T5). Each bolt in these models was assigned the calibrated connector behavior. Using this approach, the load transfer and slip mechanisms of the connection were represented without explicitly modeling local damage. The numerical results were then compared directly with experimental results to evaluate the accuracy of the simplified modeling approach in predicting the initial stiffness and ultimate capacity of more complex GFRP composite bolted connections.

### 4.1. Model Information

**Figure 19:**
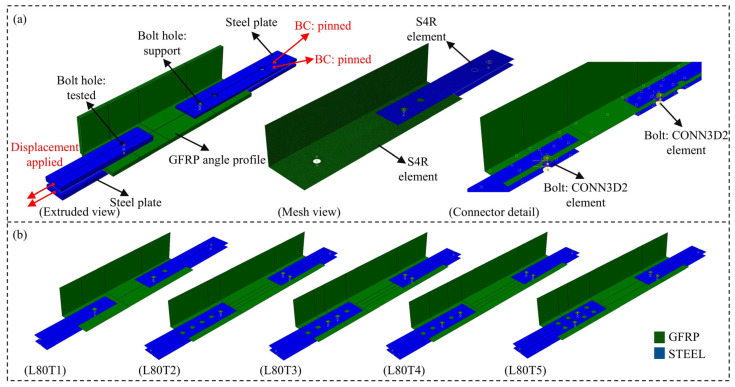
Finite element model details: (**a**) description of the model including mesh, boundary conditions, and element types; (**b**) FE model views of L80T1–T5 connections.

The general model view, mesh distribution and boundary conditions are illustrated in Figure 19a. The analyses were performed with ABAQUS/CAE 2017 [58] using a static implicit procedure with displacement-controlled loading. The GFRP angle members and steel connection plates were modeled using S4R shell elements, which offer a balance between computational efficiency and accuracy for thin-walled structures. The GFRP material was defined with the orthotropic elastic properties given in Table 8, while steel material was modeled as linear elastic isotropic with the elastic modulus and Poisson’s ratio of 200 GPa and 0.3, respectively.

**Figure 20:**
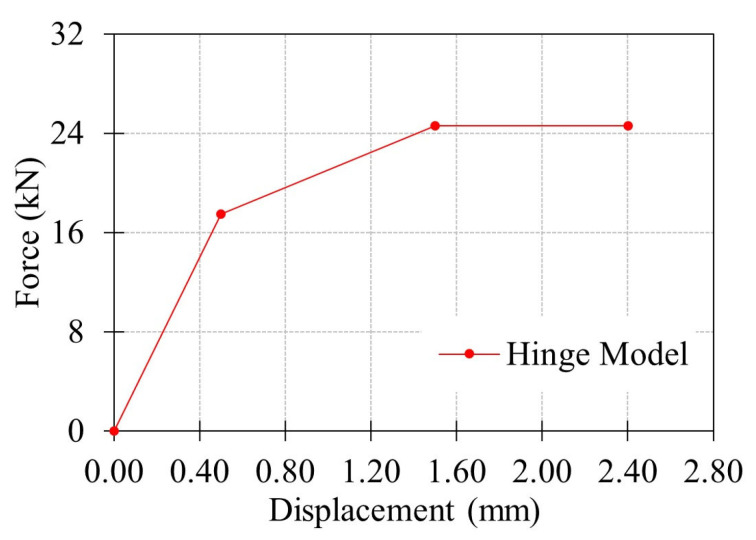
Hinge model used in connector elements for bolt modeling.

Bolt behavior was represented using CONN3D2 connector elements with a combined Cartesian–Revolute hinge formulation, as shown in Figure 20. Translational degrees of freedom were controlled through the cartesian component, while the revolute component governed rotational degrees of freedom. The hinge model was calibrated against the experimental response of the L80T1 single-bolt connection and subsequently implemented for all other configurations (L80T2–L80T5). The FE models of the respective connection configurations are presented in Figure 19b.

The interaction between the steel and GFRP members was modeled through surface-to-surface contact with normal hard contact (allowing separation) and tangential contact defined by a friction coefficient of 0.3. A mesh size of 2 mm was adopted based on mesh sensitivity studies, ensuring that the numerical results were independent of mesh refinement while maintaining computational efficiency. Using S4R shell elements for all GFRP and steel plates, the resulting models comprised 18,000–25,500 nodes and 17,500–24,500 elements. In an attempt to enable a direct comparison between test data and numerical predictions, displacements from the FE model were extracted from the same locations as the experimental LVDT measurements.

### 4.2. Results and Discussion

**Figure 21:**
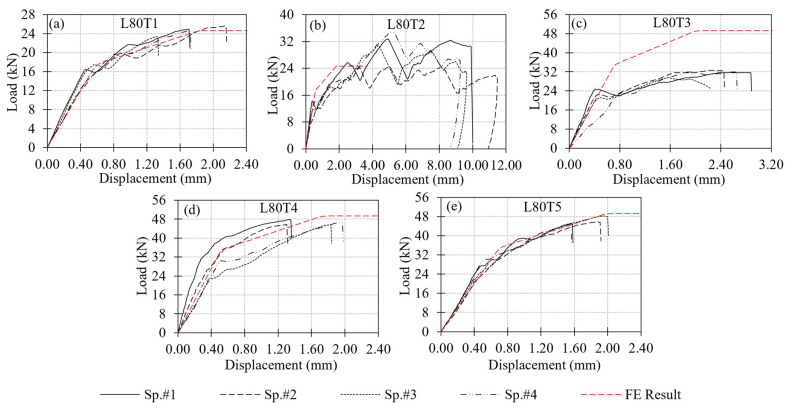
Comparison of load–displacement responses from tests and finite element simulations: (**a**) L80T1; (**b**) L80T2; (**c**) L80T3; (**d**) L80T4; (**e**) L80T5.

The numerical results were compared with the experimental load–displacement curves of the L80T1–L80T5 connections, as shown in Figure 21. Four key response parameters were used to evaluate the agreement between the FE models and the experimental tests: (i) initial stiffness, (ii) the point of stiffness reduction (onset of damage), (iii) ultimate load-carrying capacity, and (iv) displacement at failure.

For initial stiffness of the connections, close agreement is observed between the FE predictions and the experimental results across all configurations (L80T2–L80T5). This observation indicates that the simplified hinge-based model reliably captures the elastic behavior of the connections. When examining the onset of damage, ultimate capacity and displacement at failure, an acceptable agreement is valid for the L80T4 and L80T5 configurations. The FE models reproduced both the capacity levels and post-peak response of these connections with reasonable accuracy.

Different than the other connection configurations, the numerical predictions for the L80T2 and L80T3 connections show notable discrepancies with respect to the measured response. Such disagreement between the numerical and experimental responses can be attributed to the limitations of the hinge model calibration. Since the hinge formulation was calibrated based on the L80T1 single-bolt connection, which has an e/d ratio of 5, its accuracy reduces when applied to connections with different geometric constraints or governing failure modes. In testing of the L80T2 configuration, the relatively large edge distance resulted in bearing failure mechanism that is not represented in the calibration of the hinge model. In the testing of L80T3 configuration, the interaction between the two bolts that are positioned relatively close to each other (i.e., an e/d ratio of only 2.5) further reduced the prediction accuracy of the FE model.

## 5. Conclusions

The research presented in this paper addresses the development of bolted connection details for GFRP composite cross-arm members, with emphasis on their performance under realistic loading conditions in 34.5 kV energy distribution towers. As the overall performance of composite cross-arms is largely governed by the behavior of their bolted connections, the study focuses on the tensile response of bolted GFRP angle sections, examined in two experimental test groups. In Test Group 1, six pultruded GFRP angle profiles were initially considered. Single-bolt connection tests with a constant e/d of 5 were performed to examine the influence of section geometry, and the load-carrying capacity and service stiffness were determined according to ASTM D953-19, ASTM D953-10, and 1 mm hole elongation criteria. Based on the findings of Test Group 1, the L80 × 8 and L70 × 7 sections were further studied in Test Group 2 under various bolt numbers and orientations. The experimental phase of both test groups provided key insights into the governing failure modes, stiffness characteristics, and load capacity definitions. The connection capacities were also compared with predictive equations given in ASCE/SEI 74-23 to assess the conformity of the proposed connections with existing design provisions. Finally, a simplified FE model was developed with the aim of predicting important design parameters, such as load capacity and initial stiffness of GFRP bolted connections. The following conclusions are drawn from this study:

- Comparison of evaluation criteria showed that the general maximum criterion predicted the highest capacities, while the 4% hole elongation criterion was more conservative. The discrepancy between the load capacities determined based on different criteria increased with increasing connection capacity, indicating that the choice of evaluation method has a significant influence on the reported performance. Since these differences vary notably depending on the section properties, deformation-based criteria (1 mm or ASTM 4% hole elongation) are recommended for structures where deformation limits are critical to ensure a safe design. In contrast, for structures where connection deformation is not a critical design parameter, the use of the general maximum criterion may be more appropriate, as it allows for a more efficient selection of section sizes and connection designs.
- The failure mode of the connections was strongly governed by the edge distance-to-bolt diameter (e/d) ratio. Single-bolt connections with an e/d ratio of 5 exhibited initial bearing deformation around the bolt hole, followed by premature shear-out failure. Increasing the e/d ratio to 10 resulted in a dominant bearing response, accompanied by a substantial improvement in ductility. Furthermore, this transition led to a significant increase in ultimate load capacity, with improvement of 23% for L80 × 8 sections and 34% for L70 × 7 sections, demonstrating that larger edge distances effectively suppress shear-out failure while enhancing the load-carrying performance of the connection.
- The results demonstrate that the load capacity of multi-bolt GFRP connections cannot be estimated by simply summing single-bolt capacities. Connection L80T4 showed a 17% reduction, while L70T4 exhibited a 29% reduction compared to the sum of L80T1–T2 and L70T1–T2, respectively. Similarly, the capacities of L80T4 and L70T4 remained 7% and 17% lower than the expected doubled capacities of their T1 counterparts. These reductions are attributed to bolt interaction effects, which induce complex and interacting damage mechanisms under limited bolt spacing, leading to non-uniform load transfer and reduced capacity. This behavior highlights that GFRP connections require explicit consideration of interaction effects rather than linear capacity scaling.
- Shear-out strength evaluations showed that the nonwoven glass-fiber mat does not directly increase the strength, as the calculated shear-out strength remained nearly constant for all sections. The increase in load capacity is mainly due to the increase in section thickness, which provides more fiber and resin. However, sections with greater thickness and a higher number of mat layers showed improved performance under deformation-based criteria (4% and 1 mm elongation), indicating that the combined effect of increased section thickness, mat layers, and lower d/t ratio improves stress distribution around the bolt hole, delays stiffness loss, and provides a more stable post-peak response.
- The failure behavior of bolted GFRP connections is governed by a region-dependent, multi-stage mechanism in which the fiber-dominated core controls the ultimate capacity through progressive damage, whereas the mat-rich layers do not govern the ultimate strength but significantly influence the load–displacement response, damage initiation, and post-peak behavior through complex interacting mechanisms of splitting, delamination, and shear-out.
- Comparison of experimental capacities with ASCE/SEI 74-23 predictions showed that strict application of guideline-prescribed failure modes leads to generally non-conservative results, with capacity overestimations reaching up to 53% due to incorrect failure mode classification. In contrast, adopting experimentally observed failure modes improves prediction accuracy, reducing errors to 11–31%. The simplified shear-out equation provided highly consistent and conservative estimates, with prediction errors limited to 4–12%. These findings demonstrate that current e/d limits are not failure-based and are strongly influenced by material properties and bolt interaction effects, highlighting the need for mechanism-based and experimentally supported design limits for GFRP connections.
- The simplified finite element approach reproduced the connection behavior with an acceptable agreement for configurations similar to the calibration case, particularly configurations T4 and T5. However, for configurations governed by different failure mechanisms, such as T2 and T3, the model showed limited accuracy beyond the initial stiffness response. This indicates that the predictive capability of the adopted finite element approach is strongly dependent on the failure mode and the e/d ratio used in hinge model calibration. Despite this limitation, the method provides an efficient framework for modeling more complex connections with similar geometry and damage mechanisms. Furthermore, it enables the incorporation of realistic connection behavior, including load–displacement response and stiffness, into large-scale structures such as composite cross-arms in a computationally efficient and cost-effective manner, without the need for detailed damage-based modeling.

## Figures and Tables

**Table 1:** Cross-arm tip loads calculated under different loading conditions in accordance with TEİAŞ [49] specification.

Loading Combination	Factor of Safety	In-Line (kN)	Perpendicular to Line (kN)	Vertical (kN)	Nature of Loading
CL1	1.8	0	3.78	3.53	Wind Load ^1^
CL2	1.8	1.11	0	3.53	Wind Load ^1^
CL3A	1.5	5.35	0	6.49	Conductor Breakage ^2^
CL3B	1.5	0	0	8.82	Conductor Breakage ^2,3^
CL4	1.8	0	5.71	10.56	Wind Load ^2^
CL5	1.5	6.39	0	7.35	Unbalanced Icing ^2^

^1^ Wind acts in ice-free condition. ^2^ Wind acts in iced condition. ^3^ Loading condition acting on the cross-arm tips where the conductors remain connected.

**Table 2:** Load capacities of Test Group 1 specimens computed using the general maximum criteria as specified in ASTM D953-19 [54].

Section	Load Capacity (kN)						Thickness, t, Avg. ± Std. Dev. (mm)	Edge Distance, e (mm)	F_sh_ (MPa)
	Sp. #1	Sp. #2	Sp. #3	Sp. #4	Average	Std. Dev.			
L120 × 12	35.16	37.02	37.86	31.99 *	36.68	1.13	11.91 ± 0.01	53.5	28.78
L100 × 10	29.31 *	33.43	33.06	32.08	32.86	0.57	9.93 ± 0.03	53.5	30.93
L80 × 8	24.91	25.61	23.34 *	24.57	25.03	0.43	7.94 ± 0.02	53.5	29.48
L70 × 7	22.81	20.81 *	22.50	23.06	22.79	0.23	6.98 ± 0.02	53.5	30.52
L60 × 6	20.00	20.30	17.39 *	20.61	20.30	0.25	5.98 ± 0.04	53.5	31.73
L50 × 5	14.71	12.98 *	14.09	15.15	14.65	0.44	4.84 ± 0.02	53.5	28.28

* Outlier test: Avg. F_sh_ (MPa): 29.95. Std. Dev. F_sh_ (MPa): 1.21.

**Table 3:** Average load capacity and service stiffness values for Test Group 1 specimens determined using different criteria.

	Load Capacity (kN)			Service Stiffness (kN/mm)		
Section	General Maximum	1 mm Elongation	4% Elongation	General Maximum	1 mm Elongation	4% Elongation
L120 × 12	36.68 ± 1.13	29.17 ± 0.76	18.64 ± 1.66	372.25 ± 30.30	220.43 ± 16.37	217.50 ± 8.30
L100 × 10	32.86 ± 0.57	29.03 ± 1.12	18.65 ± 1.84	370.97 ± 35.21	311.77 ± 23.01	310.60 ± 30.84
L80 × 8	25.03 ± 0.43	20.17 ± 1.09	15.15 ± 0.68	317.74 ± 44.45	325.22 ± 18.35	323.60 ± 22.44
L70 × 7	22.79 ± 0.23	20.20 ± 1.04	15.52 ± 0.35	325.46 ± 17.08	333.59 ± 35.36	349.29 ± 33.72
L60 × 6	20.30 ± 0.25	20.08 ± 0.07	15.31 ± 0.79	312.06 ± 22.55	374.07 ± 38.01	380.57 ± 42.84
L50 × 5	14.65 ± 0.44	14.01 ± 1.14	10.48 ± 1.04	220.61 ± 16.70	372.99 ± 35.18	374.80 ± 39.33

**Table 4:** Average load capacities for Test Group 2 specimens determined using different criteria.

Connection Type	Load Capacity (kN)		
	General Maximum	1 mm Elongation	4% Elongation
L80T1	25.03 ± 0.43	20.17 ± 1.09	15.15 ± 0.68
L80T2	30.76 ± 3.84	14.84 ± 1.10	12.56 ± 1.56
L80T3	31.49 ± 1.22	24.17 ± 0.86	20.48 ± 4.06
L80T4	46.45 ± 0.89	39.24 ± 5.43	32.33 ± 5.20
L80T5	45.93 ± 1.34	37.25 ± 0.93	27.07 ± 2.02
L70T1	22.79 ± 0.23	20.20 ± 1.04	15.52 ± 0.35
L70T2	30.67 ± 2.25	17.79 ± 1.95	14.97 ± 0.83
L70T4	38.02 ± 3.66	29.08 ± 1.72	20.59 ± 5.39
L70T5	41.05 ± 2.13	35.21 ± 1.71	23.98 ± 1.66

**Table 5:** Observed damage modes in Test Group 1 specimens in core and mat-rich regions.

Section	GFRP Core		Mat-Rich Layers			
	Bearing Sp. #	Shear-Out Sp. #	Bearing Sp. #	Delamination Sp. #	Splitting Sp. #	Shear-Out Sp. #
L50 × 5	All	All	All	1, 4	1	1, 4
L60 × 6	All	All	All	2, 3, 4	1	3, 4
L70 × 7	All	All	All	2, 3, 4	1, 2	2, 3, 4
L80 × 8	All	All	All	1	2	1
L100 × 10	All	All	All	1, 3, 4	2, 3, 4	1, 3, 4
L120 × 12	All	All	All	3	3, 4	1, 2, 4

**Table 6:** Observed failure modes in Test Group 2 specimens for core and mat-rich regions.

Connection Type	GFRP Core		Mat-Rich Layers			
	Bearing Sp. #	Shear-Out Sp. #	Bearing Sp. #	Delamination Sp. #	Splitting Sp. #	Shear-Out Sp. #
L80T1	All	All	All	1	2	1
L80T2	All	1	All	4	1, 4	-
L80T3	-	All	-	1, 2, 4	All	-
L80T4	All	All	All	1, 4	1, 3, 4	1
L80T5	All	All	All	All	All	-
L70T1	All	All	All	2, 3, 4	1, 2	2, 3, 4
L70T2	All	2, 3, 4	All	1, 2, 4	1, 3	2, 3, 4
L70T4	All	All	All	1, 2, 3	All	1, 3
L70T5	All	All	All	All	All	1, 2, 4

**Table 7:** Comparison of experimental load capacities with predictions from ASCE/SEI 74-23 [50] provisions and simplified equations.

Connection Type	Failure ^1^	Failure ^2^	Exp. Cap. P_exp_ (kN)	Pred. Cap. P_pred_^3^ (kN)	ASCE/SEI 74-23 P_ASCE-exp_ (kN)	ASCE/SEI 74-23 P_ASCE_ (kN)	P_pred_^3^/P_exp_	P_ASCE-exp_/P_exp_	P_ASCE_/P_exp_
L80T1	Shear-out	Bearing	25.03	25.64	17.95	33.23	-	0.72	1.33
L80T2	Bearing	Bearing	30.76	33.23	33.23	33.23	-	-	-
L80T3	Shear-out	Shear-out	31.49	33.78	28.01	28.01	1.07	0.89	0.89
L80T4	Shear-out	Bearing	46.45	48.16	38.07	66.46	1.04	0.82	1.43
L80T5	Shear-out	Bearing	45.93	51.27	35.89	66.46	1.12	0.78	1.45
L70T1	Shear-out	Bearing	22.79	22.43	15.70	29.08	-	0.69	1.28
L70T2	Bearing	Bearing	30.67	29.08	29.08	29.08	-	-	-
L70T4	Shear-out	Bearing	38.02	42.14	33.31	58.15	1.11	0.88	1.53
L70T5	Shear-out	Bearing	41.05	44.87	31.41	58.15	1.09	0.77	1.42

^1^ Failure observed during the experimental testing of connection specimens. ^2^ Failure specified in ASCE/SEI 74-23 based on e/d ratio of connection specimens. ^3^ Shear-out capacity predicted by Equation (2).

**Table 8:** Material parameters of GFRP used for FE modeling.

E1 (MPa)	E2 (MPa)	E3 (MPa)	ν_12_	ν_13_	ν_23_	G12 (MPa)	G13 (MPa)	G23 (MPa)
38,400	10,400	3000	0.3	0.3	0.3	3000	3000	3000

## Data Availability

The raw data supporting the conclusions of this article will be made available by the authors on request.
